# Synthesis and Characterization of Partially Renewable Oleic Acid-Based Ionomers for Proton Exchange Membranes

**DOI:** 10.3390/polym13010130

**Published:** 2020-12-30

**Authors:** Carlos Corona-García, Alejandro Onchi, Arlette A. Santiago, Araceli Martínez, Daniella Esperanza Pacheco-Catalán, Ismeli Alfonso, Joel Vargas

**Affiliations:** 1 Instituto de Investigaciones en Materiales, Unidad Morelia, Universidad Nacional Autónoma de México, Antigua Carretera a Pátzcuaro No. 8701, Col. Ex Hacienda de San José de la Huerta, C.P. 58190 Morelia, Michoacán, Mexico; carcor93@gmail.com (C.C.-G.); alejandro.onchi@gmail.com (A.O.); ialfonso@iim.unam.mx (I.A.); 2 Escuela Nacional de Estudios Superiores, Unidad Morelia, Universidad Nacional Autónoma de México, Antigua Carretera a Pátzcuaro No. 8701, Col. Ex Hacienda de San José de la Huerta, C.P. 58190 Morelia, Michoacán, Mexico; arlette_santiago@enesmorelia.unam.mx (A.A.S.); aracelimp@enesmorelia.unam.mx (A.M.); 3 Unidad de Energía Renovable, Centro de Investigación Científica de Yucatán, A.C. Parque Científico y Tecnológico de Yucatán, Carretera Sierra Papacal-Chuburná Puerto Km 5, Sierra Papacal, 97302 Mérida, Yucatán, Mexico; dpacheco@cicy.mx

**Keywords:** oleic acid, metathesis, renewable ionomer, sulfonated polymer, long-chain polyamide, proton exchange membrane

## Abstract

The future availability of synthetic polymers is compromised due to the continuous depletion of fossil reserves; thus, the quest for sustainable and eco-friendly specialty polymers is of the utmost importance to ensure our lifestyle. In this regard, this study reports on the use of oleic acid as a renewable source to develop new ionomers intended for proton exchange membranes. Firstly, the cross-metathesis of oleic acid was conducted to yield a renewable and unsaturated long-chain aliphatic dicarboxylic acid, which was further subjected to polycondensation reactions with two aromatic diamines, 4,4′-(hexafluoroisopropylidene)bis(*p*-phenyleneoxy)dianiline and 4,4′-diamino-2,2′-stilbenedisulfonic acid, as comonomers for the synthesis of a series of partially renewable aromatic-aliphatic polyamides with an increasing degree of sulfonation (DS). The polymer chemical structures were confirmed by Fourier transform infrared (FTIR) and nuclear magnetic resonance (^1^H, ^13^C, and ^19^F NMR) spectroscopy, which revealed that the DS was effectively tailored by adjusting the feed molar ratio of the diamines. Next, we performed a study involving the ion exchange capacity, the water uptake, and the proton conductivity in membranes prepared from these partially renewable long-chain polyamides, along with a thorough characterization of the thermomechanical and physical properties. The highest value of the proton conductivity determined by electrochemical impedance spectroscopy (EIS) was found to be 1.55 mS cm^−1^ at 30 °C after activation of the polymer membrane.

## 1. Introduction

Sustainable development is commonly defined as the growth and satisfaction of current needs without compromising the scope of subsequent generations to meet their own needs [1,2,3]. Thus, affording materials in an environmentally friendly way and, consequently, reducing the use of non-renewable resources, is the main reason why the chemical industry is constantly pursuing alternatives for the synthesis of new polymers using precursors obtained from natural resources such as vegetable oils, saccharides, and biomass, among others that are available on a large scale and can be effectively used as monomers [3,4,5,6,7,8,9,10]. These renewable polymers have to be produced at low cost, with few steps for their synthesis and should also have properties comparable with their counterparts obtained from petrochemical compounds in order to be used on a commercial scale [11,12]. In this regard, vegetable oils containing unsaturated fatty acids are promising, cheap, and renewable sources to generate a huge number of oleochemicals that can be employed for the synthesis of high-value compounds in a sustainable manner [13,14]. Particularly, long-chain vegetable oils are the most important, with oleic acid being one of the most abundant fatty acids in most vegetable oils [9,14].

It is well known that olefin metathesis is a powerful and versatile tool in the petrochemical industry for various processes; however, it also has an excellent area of application in the oleochemical industry [14]. This technique provides a convenient and selective route to form dicarboxylic acids, which are basic components of great value for the current chemical industry and are used in the production of a vast variety of chemical products, among them plastics such as polyesters and polyamides [13,14,15,16]. In this context, the synthesis of polyesters derived from vanillin and fatty acid derivatives have been reported [17]; likewise, the synthesis of polyamides obtained from 1,9-azelaic acid has also been accomplished successfully [18]. Polyamides are materials considered indispensable in our daily life and are widely used for their versatile properties, such as a high melting point due to the interaction of hydrogen bridges between amide groups [19]. Today, most bio-based polyamides are aliphatic polyamides, known as nylon, and they play a vital role in industrial and commercial applications [16,18].

The quest for polymers based on renewable sources as alternatives for other highly specialized polymers is quite attractive in the polymer science since more sustainable, low-cost, and environmentally friendly routes would be available to obtain polymers whose supply is essential regardless of the depletion of fossil reserves. Nowadays, great efforts have been made to incorporate renewable sources for obtaining ionic polymers. Terpenes, tannins, lignin, pectin, keratin, gum arabic, and chitosan have been used to obtain materials with biomedical applications, food packaging, drug releasers, and wastewater treatment, among others [20,21,22]. Chitosan is the most widely used renewable raw material for obtaining ionomers for proton exchange membranes (PEMs), the good thermal and mechanical stability as well as moderate proton conductivity exhibited by the ionic polymers obtained has generated in recent years a considerable increase in the scientific literature related to this application [23,24,25,26]. These encouraging results call for the investigation and application of other sources of renewable raw materials in the chemical syntheses of these ionic polymers. For instance, the hydrophilic–hydrophobic behavior of a polyionic material aimed as a proton exchange membrane (PEM) for fuel cell applications could be subject to a more sustainable approach by incorporating long-chain aliphatic segments derived from natural compounds into the polymer structure. In this sense, it is known that the use of long-chain aliphatic dicarboxylic acids provides characteristics such as hydrophobicity, ductility, and high impact strength to the polymer backbones [27].

Based on the above, in the present study, we envisioned the use of oleic acid as an eco-friendly and renewable source to develop new ionomers intended for PEM applications. These novel oleic acid-based ionomers are aimed as a sustainable alternative to specialty synthetic polymers commonly employed as PEM and whose future availability is compromised due to the continuous depletion of fossil reserves. To accomplish this goal, we first conducted the cross-metathesis of oleic acid to yield a renewable and unsaturated long-chain aliphatic dicarboxylic acid. Then, the oleic diacid obtained was subjected to polycondensation reactions with two aromatic diamines, 4,4′-(hexafluoroisopropylidene)bis(*p*-phenyleneoxy)dianiline and 4,4′-diamino-2,2′-stilbenedisulfonic acid, as comonomers for the synthesis of a series of novel aromatic–aliphatic polyamides with increasing degree of sulfonation (DS). The polymer chemical structures were characterized by Fourier transform infrared (FTIR) and nuclear magnetic resonance (^1^H, ^13^C and ^19^F NMR) spectroscopy. Finally, the proton conductivity in membranes of these partially renewable ionomers was determined by electrochemical impedance spectroscopy (EIS). It is expected that the incorporation of the long-chain aliphatic segments will give rise to hydrophobic regions and will also impart flexibility to the ionomer membranes, whereas the sulfonated moieties will be responsible for the occurrence of the hydrophilic regions and the proton transport in the hydrated membranes. To the best of our knowledge, there have not been investigations on the use of oleic acid, a renewable vegetable oil derivative, to develop long-chain sulfonated polyamides for proton exchange membranes. Thus, this research represents an important basic scientific contribution to the green chemistry field and alternative energies as well, since it provides a more sustainable route to obtain specialty polymeric products that are used to satisfy the energy requirements of our society.

## 2. Experimental Part 

### 2.1. Characterization Techniques

^1^H-NMR, ^13^C-NMR, and ^19^F-NMR spectra were recorded on a Bruker Avance III HD at 400, 100, and 376 MHz, respectively, in DMSO-*d*_6_. Tetramethylsilane (TMS) and hexafluorobenzene (HFB) were used as internal standards. FTIR spectra were collected using a Thermo Scientific Nicolet iS10 FTIR spectrometer fitted with an attenuated total reflectance (ATR) accessory with a diamond crystal. Thirty-two spectra were obtained and coadded for each sample covering a range of 4000–650 cm^−1^ at a spectral resolution of 4 cm^−1^. Samples encapsulated in standard aluminum DSC pans were used for determining the glass transition temperature, *T*_g_, of the polymers in a TA Instruments Differential Scanning Calorimeter DSC Q2000 at a scanning rate of 10 °C min^−1^ under nitrogen atmosphere on the temperature range between 30 and 500 °C. The analysis was carried out twice. Thermomechanical analysis, TMA, at a rate of 10 °C min^−1^ under nitrogen atmosphere was performed with a TA Instruments Thermomechanical Analyzer TMA Q400 to confirm the *T*_g_ values. Thermogravimetric analysis, TGA, was conducted at a heating rate of 10 °C min^−1^ ranging from 30 to 600 °C in samples around 10 mg under nitrogen atmosphere with a TA Instruments Thermogravimetric Analyzer TGA Q5000IR in order to determine the onset of decomposition temperature, *T*_d_, of the polymers. X-ray diffraction measurements of polymer films as cast were performed on a Bruker D2-Phaser 2nd Generation diffractometer between 7 and 70 ° 2θ, at 30 KV 10 mA, using CuK_α_ radiation (1.54 Å). The density, *ρ*, of the polymers was determined in a Sartorius model Quintix 124-1s analytical balance at ambient conditions in film form employing the flotation method in ethanol. The *ρ* measurements were repeated five times at the given conditions, and the average of the values obtained for each polymer sample is reported for this parameter. The inherent viscosity, *η_inh_*, of the polymers was determined in DMSO employing a Cannon–Ubbelohde viscometer No. 50 at 30 °C at a polymer concentration of 0.2 g dL^−1^. The *η_inh_* measurements were repeated five times at the given conditions, and the average of the values obtained for each polymer sample is reported for this parameter.

The proton conductivity, *σ*, was performed using a Swagelok cell with current collectors of SS 316 using a Biologic VSP potentiostat with FRA, model VMP3B-10, from 1 MHz to 1 Hz under 100% relative humidity at 30 °C; and the values were obtained from a Nyquist plot. The mathematical expression used for calculating the proton conductivity is as follows:

(1) $$\sigma =\frac{l}{A\cdot R}\;$$

where *σ* [S cm^−1^] represents the proton conductivity, *l* is the membrane thickness [cm], *A* is the cross-section area of the membrane [cm^2^], and *R* is the resistance value of the membrane through the impedance spectroscopy [Ω].

### 2.2. Reagents

Oleic acid, 4,4′-(hexafluoroisopropylidene)bis(*p*-phenyleneoxy)dianiline (FA), and 4,4′-diamino-2,2′-stilbenedisulfonic acid (SA) were used as received. Calcium chloride (CaCl_2_), 1-methyl-2-pyrrolidinone (NMP), triphenylphosphite (TPP), and pyridine (Py) were used without further purification. Tricyclohexylphosphine [1,3-bis(2,4,6-trimethylphenyl)-4,5-dihydroimidazol-2-ylidene] [benzylidene] ruthenium dichloride (2nd Generation Grubbs catalyst) was used as received. Solvents toluene, dimethyl sulfoxide (DMSO), and methanol were used as received. All chemical reactants and solvents were acquired from Sigma-Aldrich, Inc.

### 2.3. Synthesis and Characterization of Monomer DA

**Scheme 1:**
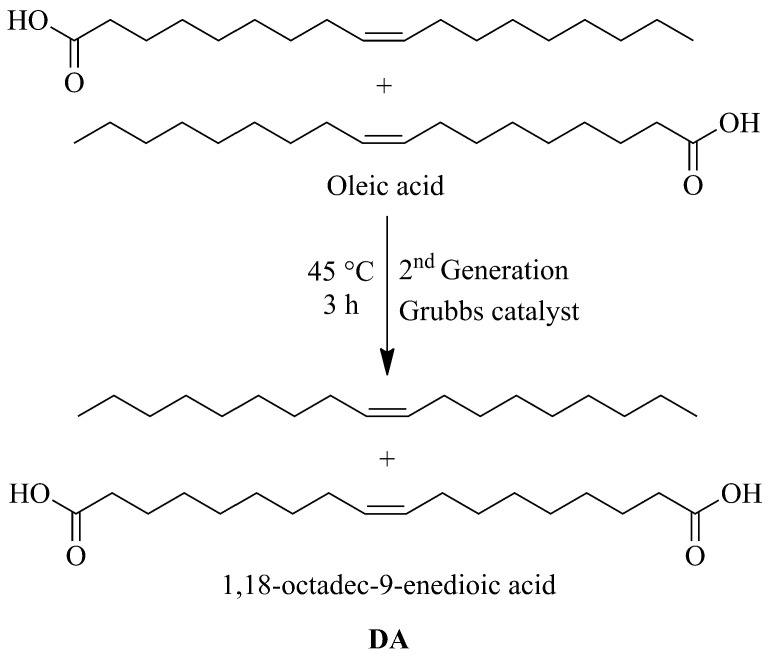
Cross-metathesis of oleic acid to yield the renewable long-chain aliphatic monomer 1,18-octadec-9-enedioic acid (**DA**).

The cross-metathesis of oleic acid to yield the monomer 1,18-octadec-9-enedioic acid (DA) was conducted as follows: 2 g (7.08 mmoL) of oleic acid and 0.006 g of 2nd Generation Grubbs catalyst were added to a 25 mL round-bottom flask equipped with a mechanical stirrer under a dry nitrogen atmosphere. The reaction mixture was heated at 45 °C for 3 h and maintained with constant stirring. Finally, the product (Scheme 1) was purified by recrystallization from toluene three times and dried under a vacuum for 24 h.

Yield = 60%. Melting point (m.p.) = 93–95 °C.

FTIR: ʋ 2918 (C−H asymmetric (asym.) str.), 2849 (C−H symmetric (sym.) str), 1694 (C=O), 1470 (C=C str.), 1427, 1408, 1317, 1259, 1289, 1220, 1190, 974, 962, 907, 730, 720, 677, 539 cm^−1^.

^1^H-NMR (400 MHz, CDCl_3_, ppm): δ 5.36 (−CH=CH−, 2H), 2.35 (−CH_2_CO−, 4H), 1.97 (−CH_2_−, 4H), 1.64 (−CH_2_−, 4H), 1.30 (−CH_2_−, 16H).

^13^C-NMR (100 MHz, CDCl_3_, ppm): δ 180.1 (C=O), 130.4 (C=C), 34.1, 32.4, 29.2, 28.9, 28.8, 28.6, 24.7.

### 2.4. Synthesis of the Polyamide Series

**Scheme 2:**
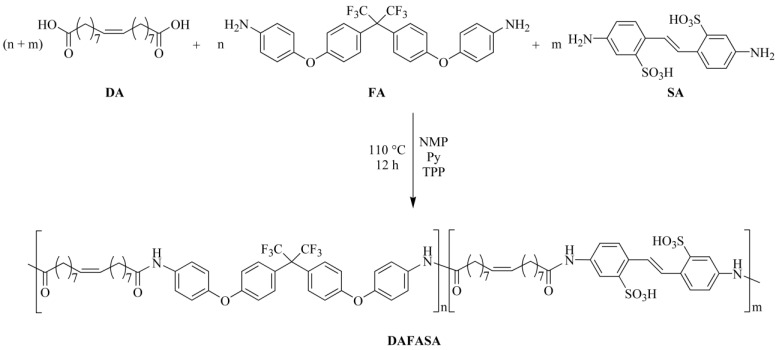
Synthesis route of the novel oleic acid-based polyamides.

A typical polymerization experiment was conducted as follows: monomer 1,18-octadec-9-enedioic acid (DA), 4,4′-(hexafluoroisopropylidene)bis(*p*-phenyleneoxy)dianiline (FA), and 4,4′-diamino-2,2′-stilbenedisulfonic acid (SA) were added to a 50 mL 3-neck flask equipped with a mechanical stirrer, under a dry nitrogen atmosphere and according to the amounts shown in Table 1. Then, 3 mL of 1-methyl-2-pyrrolidinone (NMP) and 15 wt % calcium chloride (CaCl_2_) were also added and kept under stirring for 5 min. Next, 0.41 mL of pyridine and 0.41 mL of triphenylphosphite (TPP) were added and maintained with moderate stirring until a fully incorporated mixture was achieved. The mixture was heated at 110 °C for 12 h and kept under constant stirring. Afterwards, the reaction mixture was cooled to room temperature and then precipitated into methanol. The polymer obtained (Scheme 2) was washed repeatedly with hot water in order to be purified and finally dried at 100 °C in a vacuum oven for 24 h.

#### 2.4.1. Characterization of Polymer DAFA

FTIR (thin film, cm^−1^): ʋ 3511, 3263 (N−H), 2924 (C−H asym. str.), 2853 (C−H sym. str.), 2390, 1656, 1604, 1538, 1499 (C=C str.), 1407 (C−N), 1295, 1237, 1200 (C−F), 1169, 1051, 1032, 1000, 984, 967, 914, 876, 853, 827, 734.

^1^H-NMR (400 MHz, DMSO-*d*_6_, ppm): δ 9.93 (−NH−, 2H), 7.65–7.63 (aromatic, 4H), 7.30–7.28 (aromatic, 4H), 7.06–6.98 (aromatic, 8H), 5.35 (−CH=CH−, 2H), 2.28 (−CH_2_−, 4H), 1.92 (−CH_2_−, 4H), 1.57 (−CH_2_−, 4H), 1.26 (−CH_2_−, 16H).

^13^C-NMR (100 MHz, DMSO-*d*_6_, ppm): δ 171.5 (C=O), 158.9, 150.4 (C−F), 136.6, 131.8, 130.4 (C=C), 126.4, 121.1, 120.8, 117.2, 36.8, 32.3, 29.4, 29.0, 28.8, 25.5.

^19^F-NMR (376 MHz, DMSO-*d*_6_, ppm): δ-65.9.

#### 2.4.2. Characterization of Polymer DAFASA1/4

FTIR (thin film, cm^−1^): ʋ 3291 (N−H), 2924 (C−H asym. str.), 2852 (C−H sym. str.), 1659, 1603, 1499 (C=C str.), 1406 (C−N), 1236, 1201 (C−F), 1170, 1134, 1087 (−SO_3_H, asym. str), 1015 (−SO_3_H, sym str), 966, 928, 876, 853, 828, 734, 702 (C−S).

^1^H-NMR (400 MHz, DMSO-*d*_6_, ppm): δ 9.96–9.92 (−NH−, 8H), 7.99 (aromatic, 4H), 7.71–7.69 (aromatic, 2H), 7.65–7.63 (aromatic, 12H), 7.55–7.52 (aromatic, 2H), 7.31–7.28 (aromatic, 12H), 7.06–6.99 (aromatic, 24H), 5.35 (−CH=CH−, 8H), 2.30–2.26 (−CH_2_−, 16H), 1.94–1.91 (−CH_2_−, 16H), 1.59–1.55 (−CH_2_−, 16H), 1.27 (−CH_2_−, 64H).

^13^C-NMR (100 MHz, DMSO-*d*_6_, ppm): δ 171.5 (C=O), 158.9, 150.4 (C−F), 136.6, 131.8, 130.5 (C=C), 126.3, 121.1, 120.8, 117.3, 36.7, 32.3, 29.4, 29.0, 28.8, 25.5.

^19^F-NMR (376 MHz, DMSO-*d*_6_, ppm): δ-65.9.

#### 2.4.3. Characterization of Polymer DAFASA2/4

FTIR (thin film, cm^−1^): ʋ 3295 (N−H), 2923 (C−H asym. str.), 2852 (C−H sym. str.), 1659, 1601, 1499 (C=C str.), 1406 (C−N), 1297, 1235, 1201 (C−F), 1171, 1088 (−SO_3_H, asym. str), 1036 (−SO_3_H, sym. str), 966, 928, 876, 702 (C−S).

^1^H-NMR (400 MHz, DMSO-*d*_6_, ppm): δ 10.0–9.94 (−NH−, 4H), 8.06–8.01 (aromatic, 4H), 7.73–7.71 (aromatic, 2H), 7.68–7.65 (aromatic, 4H), 7.58–7.56 (aromatic, 2H), 7.31-7.29 (aromatic, 4H), 7.04–6.99 (aromatic, 8H), 5.36 (−CH=CH−, 4H), 2.30 (−CH_2_−, 8H), 1.93 (−CH_2_−, 8H), 1.57 (−CH_2_−, 8H), 1.28 (−CH_2_−, 32H).

^13^C-NMR (100 MHz, DMSO-*d*_6_, ppm): δ 172.0, 172.0, 171.9 (C=O), 159.2, 150.7 (C−F), 146.0, 138.2, 136.9, 132.1, 130.8 (C=C), 126.9, 126.6, 121.5, 121.1, 120.1, 118.7, 117.6, 37.1, 32.7, 29.8, 29.4, 29.1, 25.9. 

^19^F-NMR (376 MHz, DMSO-*d*_6_, ppm): δ-65.9.

#### 2.4.4. Characterization of Polymer DAFASA3/4

FTIR (thin film, cm^−1^): ʋ 3296 (N−H), 2922 (C−H asym. str.), 2852 (C−H sym. str.), 1683, 1667, 1588, 1520, 1500 (C=C str.), 1488, 1464, 1435, 1397 (C−N), 1297, 1226, 1200 (C−F), 1172, 1085 (−SO_3_H, asym. str), 1027 (−SO_3_H, sym. str), 964, 901, 876, 855, 828, 706 (C−S).

^1^H-NMR (400 MHz, DMSO-*d*_6_, ppm): δ 10.0–9.95 (−NH−, 8H), 8.04–7.99 (aromatic, 12H), 7.72–7.66 (aromatic, 10H), 7.57–7.55 (aromatic, 6H), 7.32–7.27 (aromatic, 4H), 7.07–6.99 (aromatic, 8H), 5.38 (−CH=CH−), 2.29 (−CH_2_−, 16H), 1.94 (−CH_2_−, 16H), 1.58 (−CH_2_−, 16H), 1.28 (−CH_2_−, 64H).

^13^C-NMR (100 MHz, DMSO-*d*_6_, ppm): δ 171.7, 171.6 (C=O) 158.9, 150.4 (C−F), 145.8, 145.7, 137.9, 131.8, 130.5 (C=C), 126.6, 126.2, 121.2, 120.8, 119.8, 118.4, 117.3, 36.7, 32.4, 29.5, 29.1, 29.0, 28.8, 25.6.

^19^F-NMR (376 MHz, DMSO-*d*_6_, ppm): δ-65.9.

#### 2.4.5. Characterization of Polymer DASA

FTIR (thin film, cm^−1^): ʋ 3296 (N−H), 2922 (C−H asym. str.), 2851 (C−H sym. str.), 1661 (C=O), 1588, 1520 (C=C str.), 1488, 1396 (C−N), 1299, 1176, 1083 (−SO_3_H, asym. str), 1026 (−SO_3_H, sym. str), 963, 897, 818, 706.

^1^H-NMR (400 MHz, DMSO-*d*_6_, ppm): δ 10.0 (−NH−, 2H), 8.04–7.99 (aromatic, 2H), 7.99 (aromatic, 2H), 7.72–7.70 (C=C, 2H), 7.58–7.55 (aromatic, 2H), 5.38 (−CH=CH−, 2H), 2.29 (−CH_2_−, 4H), 1.95 (−CH_2_−, 4H), 1.57 (−CH_2_−, 4H), 1.28 (−CH_2_−, 16H).

^13^C-NMR (100 MHz, DMSO-*d*_6_, ppm): δ 171.7 (C=O), 145.6, 137.9, 130.5, 130.3 (C=C), 126.6, 126.2, 119.9, 118.4, 36.8, 34.1, 32.4, 29.5, 29.1, 28.8, 25.6.

### 2.5. Membrane Preparation, Ion Exchange Capacity, and Water Uptake

Membranes were cast from polymeric DMSO solutions at 60 °C. The solution was filtered and poured onto a glass plate, and the solvent was allowed to evaporate slowly under controlled DMSO atmosphere. Next, the membranes were subjected to a treatment previously described [28] consisting firstly in the removal of the residual solvent by using methanol and deionized water followed by activation with 1.0 N hydrochloric acid. Finally, the membranes were dried under a vacuum at 150 °C for 24 h. The average thickness of the films was around 400 μm.

The experimental ion exchange capacity of the polymer membrane was assessed as described in the literature [28] by the titration method using the following mathematical expression:

(2) $$IEC=\frac{V\cdot M}{W_{dry}}\;$$

where *IEC* [meq g^−1^] represents the ion exchange capacity, *V* [mL] represents the volume of NaOH solution used in the titration, *M* is the molarity of the solution, and *W_dry_* [g] is the mass of the dried membrane.

The theoretical ion exchange capacity was calculated considering a complete incorporation of monomers and according to the following expression:

(3) $$IEC_{Theo}=\frac{n_{SA}x2000}{w_{DA}+w_{SA}+w_{FA}}\;$$

where *IEC_Theo_* [meq g^−1^] represents the theoretical ion exchange capacity, *n_SA_* [mol] represents the moles of the sulfonated diamine SA, *w_DA_* [g] is the weight of monomer DA, *w_SA_* [g] is the weight of monomer SA, and *w_FA_* [g] is the weight of monomer FA.

The water uptake of the polymer membrane was estimated as described in the literature [28] by gravimetric measurements employing the expression given below:

(4) $$W_{U}=\frac{W_{wet}-W_{dry}}{W_{dry}}\cdot 100\;$$

where *W_U_* [%] represents the water uptake, *W_wet_* [g] is the mass of the hydrated membrane, and *W_dry_* [g] is the mass of the dried membrane.

## 3. Results and Discussion

The oleic acid readily underwent the cross-metathesis reaction in the presence of the 2nd Generation Grubbs catalyst affording the fully renewable monomer 1,18-octadec-9-enedioic acid (DA) in 60% yield (Scheme 1). The chemical structure of the renewable and unsaturated long-chain aliphatic monomer was successfully confirmed by FTIR and NMR, which were in agreement with previously reported data [29]. Then, partially renewable aromatic–aliphatic polyamides with an increasing degree of sulfonation were prepared successfully by polycondensation reaction employing the renewable oleic acid-based monomer DA and a mixture of two aromatic diamines as comonomers, a fluorine-containing diamine, FA, and a diamine bearing sulfonic acid groups, SA, (Scheme 2). It was found that the degree of sulfonation, DS, was effectively tailored by adjusting the feed molar ratio of these diamines. The DA monomer is considered as fully renewable because it is obtained from a raw material that is not exhausted at its source (vegetable oil fatty acid) and that can be generated again naturally over time at a speed higher than that of its consumption. In this sense, the new synthesized polyamides are considered partially renewable because they incorporate in their polymer backbones not only the renewable monomer DA but also the petroleum-based monomers FA and SA, respectively. The renewable resource content of these oleic acid-based polyamides after degradation is expected to release only the corresponding carbon to the environment, which has previously been consumed by the plant to produce the raw material. Additionally, these partially renewable polyamides could be subjected to tertiary or chemical recycling by depolymerization reactions in order to try recovering the renewable materials, which includes hydrolysis, alcoholysis, and dry-heat processes, although mechanical recycling is actually regarded as the most sustainable alternative [30]. Polymers obtained from renewable raw material sources biodegrade under controlled conditions, for example under composting conditions or through fermentation. In this context, the biodegradability of an oleic acid-based compound has been recently assessed by the biochemical oxygen demand (BOD) method using activated sludge and found to be readily biodegradable [31].

**Figure 1:**
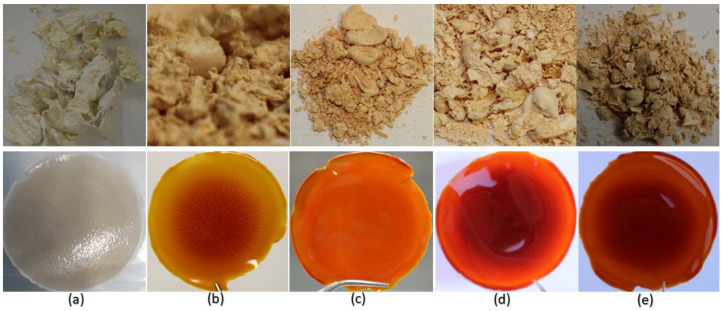
Photographic images of the synthesized raw oleic acid-based polyamides (top) and their corresponding polymeric membranes (bottom): (**a**) DAFA, (**b**) DAFASA1/4, (**c**) DAFASA 2/4, (**d**) DAFASA 3/4, and (**e**) DASA.

Figure 1 shows the photographic images of the raw oleic acid-based polyamides synthesized in this study as well as the membranes prepared from such partially renewable polymers. It can be seen that the nonsulfonated polyamide (Figure 1a) yielded white fibers, while the sulfonated polyamides (Figure 1b–e) afforded fibers with a coloration ranging from slightly orange to pale brown, whose size became smaller as the concentration of the −SO_3_H groups increased. From solutions of long-chain polyamides in DMSO, polymer membranes were prepared, which were transparent in appearance and quite resistant to touch when the membrane lacks sulfonic acid groups, and opaque with an orange color that intensified progressively from a light color to a darker one as the DS increased. The membrane rigidity of the sulfonated polyamide series increased with the DS, being fairly flexible under hydration conditions but a little brittle in the dry state. Sulfonated membrane samples (100–150 mg) were used to determine the hydrolytic stability of the ionomers. To accomplish this, we conducted aging experiments by immersing the ionomer membranes in 500 mL of deionized water at 90 °C up to 72 h. The membranes showed pretty good hydrolytic stability, since neither toughness detriment nor signs of dissolution were observed under these conditions.

**Figure 2:**
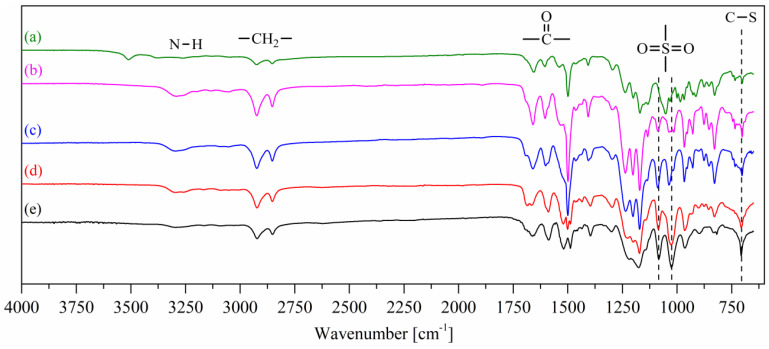
Fourier transform infrared (FTIR) spectra of the oleic acid-based polyamides: (**a**) DAFA, (**b**) DAFASA1/4, (**c**) DAFASA2/4, (**d**) DAFASA3/4 and (**e**) DASA.

FTIR spectroscopy was performed to confirm the chemical structures of the novel oleic acid-based polyamides; the FTIR spectra of these novel polymers are compared in Figure 2. The characteristic absorption band for the N−H bond is observed around 3300 cm^−1^, the absorption bands due to the antisymmetric and symmetric stretch tension of the aliphatic moiety are shown around 2924 cm^−1^ and 2852 cm^−1^, respectively, the band associated to the amide carbonyl groups (−CO−NH−) is displayed near 1660 cm^−1^, the absorption band of the C=C is exhibited around 1500 cm^−1^, and the band attributed to the C−F is seen about 1200 cm^−1^. The presence of the sulfonic acid groups in the polymer backbone is indicated by the absorption bands shown about 1086 and 1007 cm^−1^, which were ascribed correspondingly to the asymmetric and symmetric O=S=O stretching vibrations of the −SO_3_H groups, while those signals appearing around 660 cm^−1^ are attributed to the C−S stretching vibration in the sulfonated polyamides, respectively. A gradual increment in the last three mentioned bands is observed as the degree of sulfonation is also increased.

**Figure 3:**
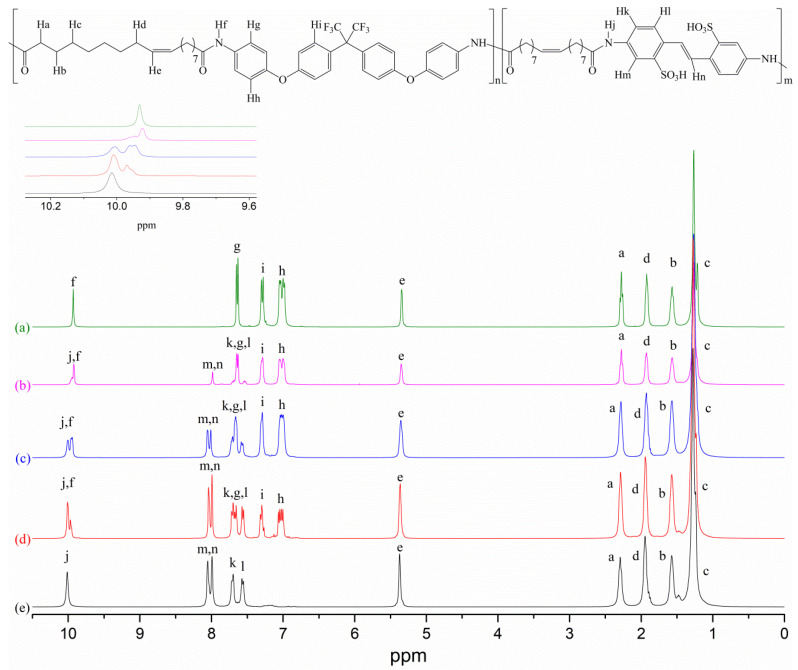
^1^H-NMR spectra of the long-chain polyamides: (**a**) DAFA, (**b**) DAFASA1/4, (**c**) DAFASA2/4, (**d**) DAFASA3/4 and (**e**) DASA.

NMR spectroscopy was performed to confirm the chemical structures and the compositions of the novel oleic acid-based polyamides. The ^1^H NMR spectra of this polyamide series are compared in Figure 3. This analysis indicates that the relationship of the proton integration areas agrees quite well with the expected long-chain polyamide structures. For all of the polymers (Figure 3a–e), the signals attributed to the aliphatic moiety resulting from the incorporation of the monomer DA in the polymeric backbones are observed in the range of δ = 2.6–1.1 ppm corresponding to the protons (H_a_, H_b_, H_c_, and H_d_) of the methylene groups, whereas the signal ascribed to the olefinic protons (H_e_) appears around δ = 5.36 ppm. For the DAFA polymer (Figure 3a), the signal corresponding to the characteristic amide proton, H_f_, appears at 9.93 ppm, while the signals ascribed to the aromatic protons H_g_, H_i_, and H_h_ resulting from the incorporation of the fluorinated diamine FA in the polyamide are seen in the range of 7.65 to 6.99 ppm. For the DASA polymer (Figure 3e), the signal corresponding to the characteristic amide proton, H_j_, appears at 10.0 ppm, while the signals ascribed to the aromatic protons H_m_, H_n_, H_k_, and H_l_ resulting from the incorporation of the sulfonated diamine SA in the polyamide are observed in the range of 8.04 to 7.56 ppm.

For the rest of the copolyamides in the series (Figure 3b–d), two peaks corresponding to the protons found in the –NH– moieties mentioned above are observed. Likewise, all the signals attributed previously to the aromatic protons of both diamines are also observed, and their intensities vary progressively in accordance with the feed molar ratio of these diamines. The relationship between the proton integration areas that remain unchanged in the polyamide DAFA, the polyamide DASA, and the series of sulfonated polyamides DAFASA, H_h_, H_i_, H_m_, and H_n_, was employed to calculate the DS. The values of DS determined from the ^1^H NMR spectra for the long-chain polyamides DAFASA1/4, DAFASA2/4, and DAFASA3/4 were found to be 23.7%, 47.3%, and 71.6%, respectively, which agree with the expected values from the monomer feed molar ratios (25%, 50%, and 75%, respectively). These outcomes indicate that the amount of the –SO_3_H groups intended for the macromolecular architectures of these renewable polymers was effectively controlled by varying the feed molar ratio of FA to SA diamines.

**Figure 4:**
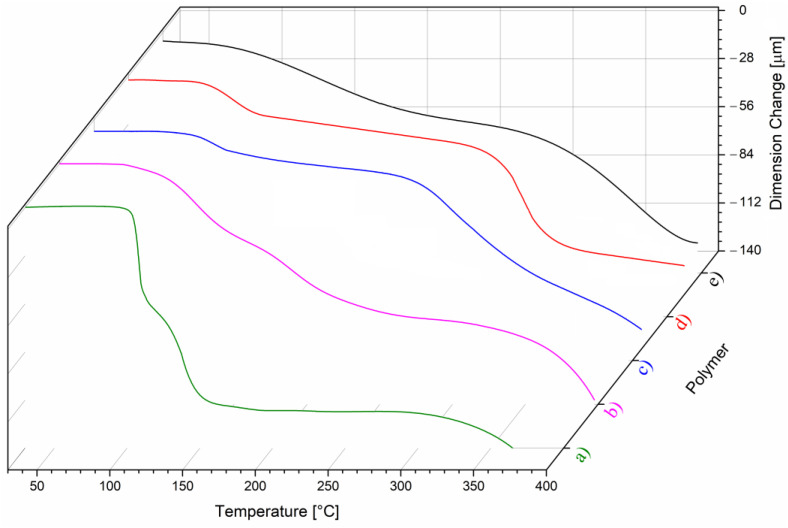
Thermomechanical curves (TMA) of the long-chain polyamides: (**a**) DAFA, (**b**) DAFASA1/4, (**c**) DAFASA2/4, (**d**) DAFASA3/4 and (**e**) DASA.

The physical properties of the partially renewable long-chain polyamide series are summarized in Table 2. For all the polymers synthesized, two glass transition temperatures, *T*_g_, were detected by TMA (Figure 4). The first one, *T*_g1_, ranging from 96 °C to 105 °C, is attributed to the highly flexible aliphatic moiety resulting from the incorporation of monomer DA in the polymer backbone, whereas the second one, *T*_g2_, ranging from 131 to 319 °C, is ascribed to the rigid aromatic moieties of the polymer main chain. It was found that for the nonsulfonated polymer DAFA, *T*_g2_ is lower than those of the other polyamides bearing −SO_3_H groups, since ether linkages from the fluorinated diamine, FA, impart proportionally higher segmental mobility to the polymer main chain. In contrast, for the sulfonated polyamides, *T*_g2_ increased as the DS was also increased, which indicates that *T*_g2_ is being affected by the introduction of −SO_3_H groups in the macromolecular architecture that causes two effects associated to the inhibition of the relaxation process of the polymer chains that in turn increase their stiffness. On one hand, the conformational mobility of the polymer chains is diminished, since the ionic character of the sulfonic acid group heightens the interactions between polymer chains. On the other hand, the presence of the −SO_3_H groups causes considerable steric effects to the polymer backbone that hinder the torsional movement of the macromolecules. Other sulfonated polymers have also shown these effects [28,32].

**Figure 5:**
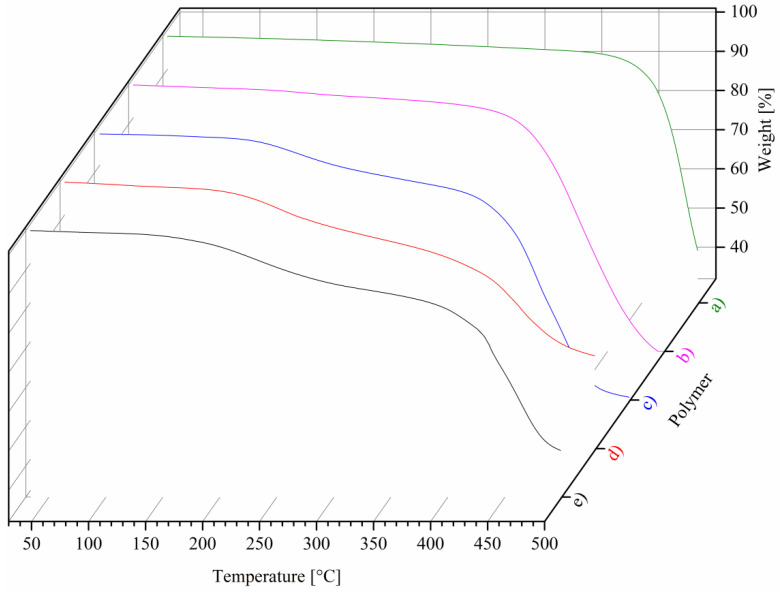
Thermogravimetric curves (TGA) of the long-chain polyamides: (**a**) DAFA, (**b**) DAFASA1/4, (**c**) DAFASA2/4, (**d**) DAFASA3/4, and (**e**) DASA.

TGA was employed to study the thermal stability of the oleic acid-based polyamides under N_2_ atmosphere, and the thermograms are presented in Figure 5. From this analysis, a single onset temperature for decomposition, *T*_d_, about 457 °C for the nonsulfonated polyamide DAFA was observed and associated to the initial degradation of the aromatic polymer backbone. This polymer exhibits the highest thermal stability as compared with the sulfonated polyamides and could be mainly attributed to the incorporation of the fluorine-containing moieties in the polymer. This enhancement in the thermal stability of the polymer when the fluorinated diamine FA is incorporated in the macromolecular structure has been previously attributed to the difference in bond energies of C−F and C−H as well as the relative number of aromatic groups per repeat unit of the polymer main chain [33]. It is worth noting that all the sulfonated polyamides show two weight losses during the thermal degradation. The first one, *T*_d1_, detected in the range of 155 to 197 °C is ascribed to the loss of the −SO_3_H groups bonded to the aromatic moieties. As it can be seen, the more content of −SO_3_H groups in the sulfonated polymer, the higher the decomposition temperature afforded. The second decomposition stage, *T*_d2_, of the ionomers was seen in the range of 385 to 423 °C and attributed to the thermal degradation of the polymer main chain. Other sulfonated polymers have also shown this behavior [34].

Polymer films from the novel long-chain polyamides were subjected to X-ray diffraction (XRD) to estimate the degree of crystallinity, *DC*, as well as the mean intersegmental distance or *d*-spacing value between polymer chains by applying the Bragg’s equation

**Figure 6:**
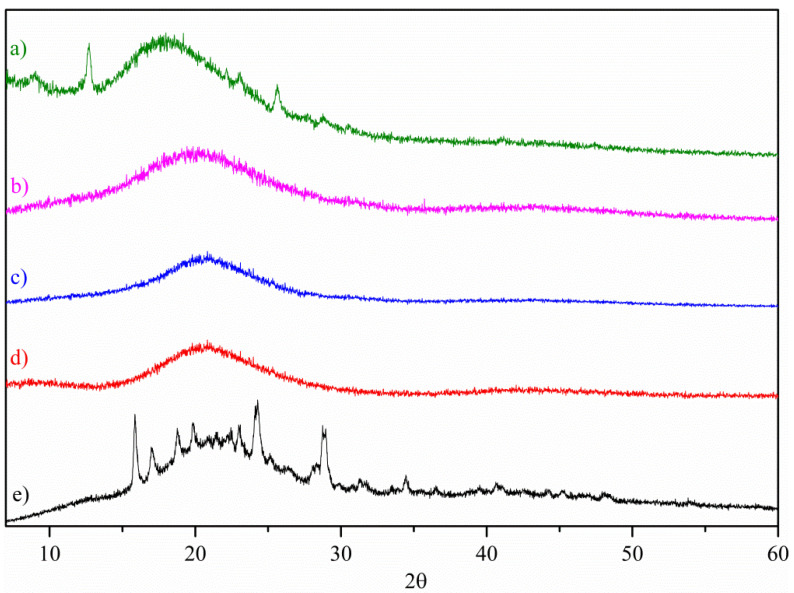
X-ray diffraction patterns of the long-chain polyamides: (**a**) DAFA, (**b**) DAFASA1/4, (**c**) DAFASA2/4, (**d**) DAFASA3/4, and (**e**) DASA.

(5) nλ = 2dsinθ

at the angle of maximum height of the amorphous peak [28]. In general, the XRD measurements performed on the films revealed amorphous materials with diffraction patterns displaying one broad peak that is characteristic of this kind of polymer and whose maximum reflective intensity was observed at 2θ ≈ 20° (Figure 6). For polyamides DAFA and DASA, small regions of crystallinity accounting for 5.3% and 12.8%, respectively, were detected and mainly attributed to the aromatic ring stacking in the former and to the strong electrostatic attraction generated by the −SO_3_H groups in adjacent polymer chains in the latter. No degree of crystallinity for the remaining sulfonated polyamides of the series was detected, suggesting that the combination of fluorinated and sulfonated moieties hinders the polymer chain packing, thus leading to completely amorphous materials. In addition, from Table 2, it is observed that the *d*-spacing value diminishes gradually as the DS in the polymer backbone increases, which is associated to the increased polymer interchain interaction induced by the ionic character of the −SO_3_H groups.

The density, *ρ*, of the novel polyamides was determined at ambient conditions in film form employing the flotation method in ethanol. The values, shown in Table 2, were found to be ranging from 1.24 to 1.27 g cm^−3^, and they indicate that a decrease in the packing efficiency of polymer chains is promoted after the incorporation of sulfonated moieties in the long-chain polyamides, which could be attributed to the high molecular bulkiness of the −SO_3_H group. The latter results in lower values of the polymer density along with higher values of the fractional free volume, *FFV*, in comparison with those values found for the nonsulfonated polyamide DAFA. The Bondi group contribution method [35] was applied to calculate the *FFV* according to the following mathematical expression:

(6) $$FFV=\left(V-V_{0}\right)/V\;$$

where V is the specific volume of the polymer (1/ρ), *ρ* is the polymer density [g cm^−3^], $V_{0}$ is the occupied volume calculated as $V_{0\;}=\;1{.3\;V}_{w}$, where $V_{w}$ represents the van der Waals volume estimated from the data tabulated in van Krevelen [36].

As listed in Table 2, the inherent viscosities of the oleic acid-based polyamides were in the range from 0.33 to 0.37 dL g^−1^, suggesting the formation of relatively high molecular weights that allowed obtaining tough films after the casting–heating stage. It is seen that as the concentration of −SO_3_H groups in the polymer increased, the inherent viscosity was decreased. This trend has been also found in other polyamides bearing sulfonic acid groups [37].

Table 3 summarizes the ionic properties of the novel oleic acid-based polyamides synthesized in this study. It is observed that the membrane prepared from the nonsulfonated polyamide DAFA exhibits the lowest water uptake, *W_u_* = 1.9%, of all the membranes obtained. This value is considerably heightened as the DS raises due to the presence of more hydrophilic regions promoted by an increasing number of sulfonic acid groups in the polymer structure. In this regard, the ionomer containing the lowest content of −SO_3_H groups, polyamide DAFASA1/4, exhibited a water uptake of 9.7%, whereas that ionomer bearing the highest one, polyamide DASA, showed a *W_u_* of 44.1%. This improvement in the water absorption of the ionomer is a feature highly desirable for PEM applications. The ion exchange capacity, *IEC*, of the sulfonated polymers in film form was determined experimentally by titration with a NaOH solution at room temperature. It was found that the *IEC* increased with the progressive introduction of sulfonic acid groups in the macromolecule. This parameter provides an estimation of the amount of functional groups able to be ionized in the polymer membrane, which are largely responsible for the efficiency in the proton conductivity of a PEM. Thereby, the experimental *IEC* values determined for the polymer membranes ranged from 0.48 to 2.28 meq g^−1^, respectively, and they agree with the theoretical *IEC* values also shown in Table 3.

**Figure 7:**
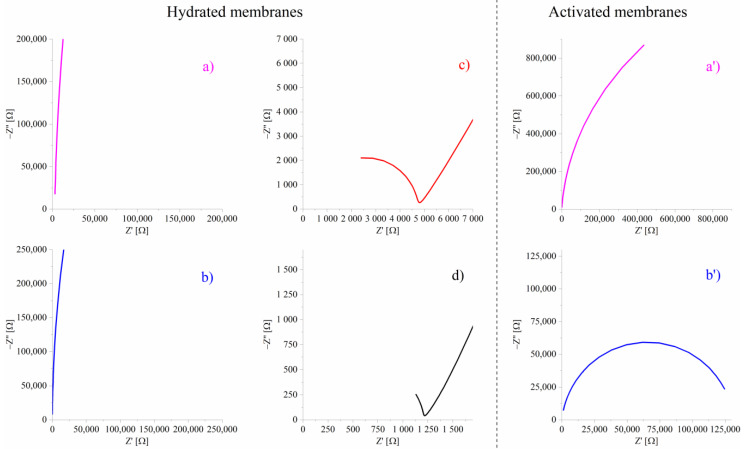
Nyquist plots for the oleic acid-based ionomers; before activation with HCl (left): (**a**) DAFASA1/4, (**b**) DAFASA2/4, (**c**) DAFASA3/4 and (**d**) DASA, and after activation with HCl (right): (**a′**) DAFASA1/4, (**b′**) DAFASA2/4, respectively.

The electrochemical impedance spectroscopy (EIS) measurements of the membranes DAFASA1/4, DAFASA2/4, DAFASA3/4, and DASA were performed in the range of 1 MHz to 1 Hz under 100% relative humidity at 30 °C. Figure 7 shows the Nyquist plots for the oleic acid-based ionomer membranes. From the complex plane plot Z″ vs. Z′, where Z′ represents the real part of impedance and Z′′ represents the imaginary part of impedance, the intersection of the arc with the abscissa axis at high frequencies gives the ohmic resistance of the membrane to proton transport. In this regard, the resistance, *R*, of the membranes was calculated using the Circle Fit function of the EC-Lab V11.27 software, and all measures were repeated three times at the given conditions, and the averages of the results obtained are reported. Then, the proton conductivity, *σ*, was calculated in accordance with Equation (1), and the results are presented in Table 3. Firstly, the EIS measurements were carried out successfully in the hydrated membranes of all the sulfonated polyamide series before being activated with 1.0 N HCl. It was found that the proton conductivity increased as the degree of sulfonation of the polyamide was also increased. Other sulfonated polymers have also shown this effect [38]. In this sense, the polyamide DAFASA1/4 bearing the lowest concentration of −SO_3_H groups afforded a *σ* value of 0.035 mS cm^−1^, while the highly sulfonated polyamide DASA accounted for a *σ* value of 0.594 mS cm^−1^. Then, EIS measurements were conducted in the ionomer membranes after being activated with hydrochloric acid solution. The results show that the *σ* value in the activated membrane of polyamide DAFASA1/4 is almost ten times larger than that of the nonactivated membrane. Similarly, the *σ* value of polyamide DAFASA2/4 was increased from 0.407 to 1.552 mS cm^−1^ after the activation process.

Unexpectedly, the membranes DASA and DAFASA3/4 could not be assessed in the activated form because they did not withstand the activation procedure with the HCl solution, which caused the membranes to lose their mechanical stabilities, making their handling challenging and finally leading to the membrane cracking. It is likely that the highly acidic medium that is generated during the proton exchange in the polyamide membranes with higher degrees of sulfonation affects the carbon–carbon double bonds of the polymer main chain, which in turn could be reflected in a decrease of the molecular weight; more research is needed in order to clarify this issue. For instance, in order to circumvent this drawback, these renewable ionomers could undergo chemical cross-linking at the carbon–carbon double bonds, thus enhancing the membrane mechanical and chemical resistance. An effective approach to perform this chemical modification via metathesis [33] without significantly affecting the solubility of the resulting polymer has been recently reported, and its use to cross-link these long-chain polyamides could be the subject of a forthcoming paper.

**Figure 8:**
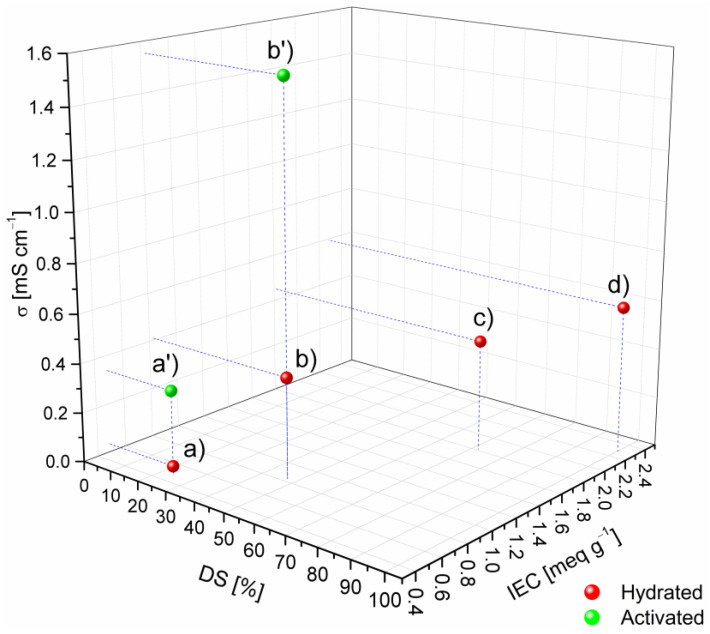
*IEC* and *σ* as function of degree of sulfonation (DS) for the polymers; before activation with HCl: (**a**) DAFASA1/4, (**b**) DAFASA2/4, (**c**) DAFASA3/4 and (**d**) DASA, and after activation with HCl: (**a′**) DAFASA1/4, (**b′**) DAFASA2/4, respectively.

Figure 8 shows graphically the relationship that prevails among the ionic parameters assessed in the long-chain sulfonated polyamide membranes. It is clearly observed that the *IEC* and the proton conductivity increase together with the degree of sulfonation and that the membrane activation step conducted on the oleic acid-based ionomer membranes is also reflected in higher values of *σ*.

Table 4 compiles the ionic properties in different sulfonated polymers previously reported in the literature and compared with those found in the oleic acid-based ionomers synthesized in the present study. As it is seen, the results obtained for polymers DASA and DAFASA2/4 are comparable with respect to other fully synthetic polymers bearing sulfonic acid groups. It should be noted that these outcomes are quite close to those reported for some sulfonated polyimides, which are materials commonly intended for PEM applications. The foregoing reveals that the renewable approach proposed in this work is an effective tool for developing sustainable and eco-friendly specialty polymers.

## 4. Conclusions

Oleic acid underwent effectively cross-metathesis to yield a renewable and unsaturated long-chain aliphatic dicarboxylic acid (DA), which was successfully subjected to polycondensation reactions with two aromatic diamines as comonomers for the synthesis of a series of partially renewable aromatic-aliphatic polyamides with an increasing degree of sulfonation (DS). FTIR and NMR spectroscopy confirmed the polymer chemical structures and also revealed that the DS was effectively tailored by adjusting the feed molar ratio of the diamines. TMA showed the occurrence of two glass transition temperatures in all the long-chain polyamides. The first one was attributed to the highly flexible aliphatic moiety resulting from the incorporation of monomer DA in the polymer backbone, whereas the second one was ascribed to the rigid aromatic moieties of the polymer main chain. It was also found that the water uptake, the ion exchange capacity, and the proton conductivity (*σ*) increase together with the DS, and that the membrane activation step conducted on the partially renewable ionomer membranes is also reflected in higher values of *σ*. The highest value of *σ* determined by electrochemical impedance spectroscopy (EIS) was found to be 1.55 mS cm^−1^ at 30 °C after activation of the polymer membrane. The partially renewable oleic acid-based ionomers reported in this study exhibit ionic properties quite close to those of some of the fully synthetic sulfonated polymers previously reported in the literature, indicating that the renewable approach proposed in this work is an effective tool for developing sustainable and eco-friendly specialty polymers intended for PEM applications.

## Figures and Tables

**Table 1:** Molar relations of monomers in the feed for the synthesis of the long-chain polyamide series.

Polymer	DA ^a^ [n+m, mmol]	FA ^b^ [n, mmol)]	SA ^c^ [m, mmol]
DAFA	0.64	0.64	0
DAFASA1/4	0.64	0.48	0.16
DAFASA2/4	0.64	0.32	0.32
DAFASA3/4	0.64	0.16	0.48
DASA	0.64	0	0.64

^a^ 1,18-octadec-9-enedioic acid. ^b^ 4,4′-(hexafluoroisopropylidene)bis(*p*-phenyleneoxy)dianiline. ^c^ 4,4′-diamino-2,2′-stilbenedisulfonic acid.

**Table 2:** Physical properties of the partially renewable long-chain polyamide series.

Polymer	*T*_g_ [°C] ^a^	*T*_d_ [°C] ^b^	*DC* [%] ^c^	*d_interchain_* [Å] ^d^	*ρ* [g cm^−3^] ^e^	*FFV* [%] ^f^	*η_inh_* [dL g^−1^] ^g^
DAFA	105 ^h^/131 ^i^	456	5.3	4.93	1.271	13.7	0.374
DAFASA1/4	103 ^h^/175 ^i^	155 ^j^/385 ^k^	ND ^l^	4.56	1.241	15.7	0.369
DAFASA2/4	100 ^h^/248 ^i^	176 ^j^/388 ^k^	ND ^l^	4.38	1.247	15.2	0.361
DAFASA3/4	96 ^h^/286 ^i^	194 ^j^/406 ^k^	ND ^l^	4.25	1.249	15.1	0.356
DASA	96 ^h^/319 ^i^	197 ^j^/423 ^k^	12.8	4.1	1.245	15.3	0.336

^a^ Glass transition temperature determined by TMA. ^b^ Decomposition temperature determined by TGA. ^c^ Degree of crystallinity determined by XRD. ^d^ Average separation distance between polymer chains determined by XRD. ^e^ Density determined by the flotation method. ^f^ Fractional free volume calculated by the Bondi group contribution method. ^g^ Inherent viscosity at a polymer concentration of 0.2 g dL^−1^. ^h^ Temperature (*T*_g1_) ascribed to the glass transition that undergo the flexible aliphatic moieties. ^i^ Temperature (*T*_g2_) ascribed to the glass transition that undergo the rigid aromatic moieties. ^j^ Temperature (*T*_d1_) attributed to the decomposition of sulfonic acids groups. ^k^ Temperature (*T*_d2_) attributed to the decomposition of the polymer backbone.

**Table 3:** Ionic properties of the partially renewable oleic acid-based polyamide series.

Polymer	*DS* [%] ^a^	*W_U_* [%] ^b^	*IEC_Theo_* [meq g^−1^] ^c^	*IEC* [meq g^−1^] ^d^	*σ* Hydrated [mS cm^−1^] ^e^	*σ* Activated [mS cm^−1^] ^e^
DAFA	0	1.9	NA ^f^	NA ^f^	NA ^f^	NA ^f^
DAFASA1/4	23.7	9.7	0.63	0.482	0.035	0.34
DAFASA2/4	47.3	17.1	1.321	0.832	0.407	1.552
DAFASA3/4	71.6	36.4	2.084	1.816	0.449	Pending ^g^
DASA	100	44.1	2.929	2.287	0.594	Pending ^g^

^a^ Degree of sulfonation determined by ^1^H NMR. ^b^ Water uptake determined by gravimetric measurements. ^c^ Theoretical ion exchange capacity calculated according to Equation (3). ^d^ Experimental ion exchange capacity determined by titration with 0.01 M NaOH. ^e^ Proton conductivity determined by electrochemical impedance spectroscopy (EIS). ^f^ Not applicable. The membrane lacks −SO_3_H groups to conduct the measurement. ^g^ The membrane did not withstand the activation process.

**Table 4:** Comparison of the ionic properties of the partially renewable ionomers reported in this study and some polymers reported in the literature.

Polymer	*IEC* Experimental [meq g^−1^] ^a^	*W_U_* [%] ^b^	*σ* [mS cm^−1^] ^c^	Reference Author
C5FNDDIH5 ^d^	0.3	37.3	0.1	[39]
Poly-SHPhNDI ^e^	0.8	12.4	0.4	[32]
DASA	2.2	44.1	0.5	This study
CH3 5 30/70 ^f^	0.9	19	1.3	[40]
DAFASA2/4	0.8	17.1	1.5	This study
CF3 5 30/70 ^g^	0.8	14	1.7	[41]
HFAS55 ^h^	1.6	22.5	3.3	[37]
CMV ^i^	2.4	25	5.1	[42]

^a^ Ion exchange capacity determined by titration. ^b^ Water uptake determined by gravimetric measurements. ^c^ Proton conductivity determined by electrochemical impedance spectroscopy (EIS). ^d^ Sulfonated copolynorbonene dicarboximide. ^e^ Sulfonated polynorbonene dicarboximide. ^f^ Sulfonated naphthalenic polyimide. ^g^ Sulfonated naphthalenic fluoropolyimide. ^h^ Sulfonated copolyamide. ^i^ Sulfonated polyarylene.

## Data Availability

The data presented in this study are available on request from the corresponding author.
